# Chronotherapeutic neuroprotective effect of verapamil against lipopolysaccharide-induced neuroinflammation in mice through modulation of calcium-dependent genes

**DOI:** 10.1186/s10020-022-00564-8

**Published:** 2022-11-26

**Authors:** Esraa M. Mosalam, Aya Ibrahim Elberri, Amany Said Sallam, Heba Rady Salem, Ebtehal M. Metwally, Mahmoud S. Abdallah, Moataz A. Shaldam, Hend E. Abo Mansour

**Affiliations:** 1 Biochemistry Department, Faculty of Pharmacy, Menoufia University, Shebin El-Kom, 32511 Menoufia Egypt; 2 Genetic Engineering and Molecular Biology Division, Department of Zoology, Faculty of Science, Menoufia University, Shebin El-Kom, 32511 Menoufia Egypt; 3 Department of Pharmacology and Toxicology, Faculty of Pharmacy, Menoufia University, Shebin El-Kom, 32511 Menoufia Egypt; 4 Medical Physiology Department, Faculty of Medicine, Menoufia University, Shebin El-Kom, 32511 Menoufia Egypt; 5 Clinical Pharmacy Department, Faculty of Pharmacy, University of Sadat City (USC), Sadat City, 32897 Egypt; 6 Department of Pharmaceutical Chemistry, Faculty of Pharmacy, Kafrelsheikh University, Kafrelsheikh, Egypt

**Keywords:** Alzheimer’s disease, Lipopolysaccharide, Verapamil, Calcium, CAMKII, Chronotherapy

## Abstract

**Background:**

Neuroinflammation is a major mechanism in neurodegenerative diseases such as Alzheimer’s disease (AD), which is a major healthcare problem. Notwithstanding of ample researches figured out possible molecular mechanisms underlying the pathophysiology of AD, there is no definitive therapeutics that aid in neuroprotection. Therefore, searching for new agents and potential targets is a critical demand. We aimed to investigate the neuroprotective effect of verapamil (VRP) against lipopolysaccharide (LPS)-induced neuroinflammation in mice and whether the time of VRP administration could affect its efficacy.

**Methods:**

Forty male albino mice were used and were divided into normal control, LPS only, morning VRP, and evening VRP. Y-maze and pole climbing test were performed as behavioral tests. Hematoxylin and eosin together with Bielschowsky silver staining were done to visualize neuroinflammation and phosphorylated tau protein (pTAU); respectively. Additionally, the state of mitochondria, the levels of microglia-activation markers, inflammatory cytokines, intracellular Ca^2+^, pTAU, and Ca^2+^-dependent genes involving Ca^2+^/ calmodulin dependent kinase II (CAMKII) isoforms, protein kinase A (PKA), cAMP response element-binding protein (CREB), and brain-derived neurotrophic factor (BDNF), with the level of VRP in the brain tissue were measured.

**Results:**

LPS successfully induced neuroinflammation and hyperphosphorylation of tau protein, which was indicated by elevated levels of microglia markers, inflammatory cytokines, and intracellular Ca^2+^ with compromised mitochondria and downregulated CAMKII isoforms, PKA, CREB and BDNF. Pretreatment with VRP showed significant enhancement in the architecture of the brain and in the behavioral tests as indicated by the measured parameters. Moreover, morning VRP exhibited better neuroprotective profile compared to the evening therapy.

**Conclusions:**

VRP highlighted a multilevel of neuroprotection through anti-inflammatory activity, Ca^2+^ blockage, and regulation of Ca^2+^-dependent genes. Furthermore, chronotherapy of VRP administration should be consider to achieve best therapeutic efficacy.

**Graphical Abstract:**

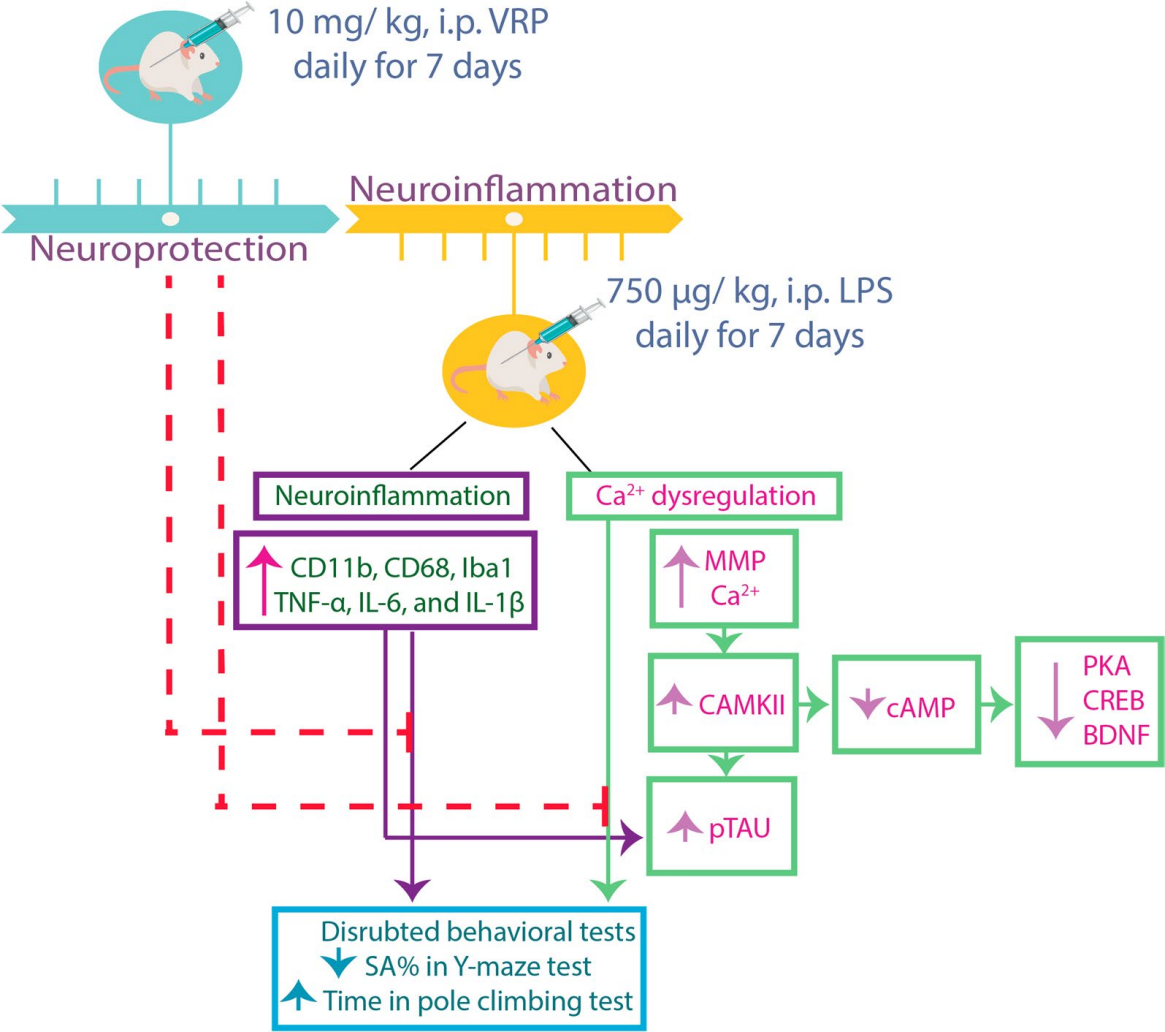

## Background

Neuroinflammation is a major culprit in neurodegenerative diseases such as Alzheimer’s disease (AD) (Batista et al. 2019). AD usually starts with no clearly identified symptoms until neuronal damage occurs and that may happen over years (Barthélemy et al. 2020). Patients with AD manifest impaired cognition, deficit memory, changed behavioral and emotional states, and later they may become unable to do the basics of life involving walking, eating, and other activities. AD accounts for 60–80% of overall cases of dementia (Alzheimer’s disease facts and figures 2021) with a prevalence of 10–30% and 1–3% incidence rate in people older than 65 years (Masters et al. 2015).

Tremendous researches have been done to explore the underlying mechanisms that are implicated in the development of AD and they have suggested multiple hypotheses such as accumulation of amyloid beta (Aβ), neurofibrillary tangles (NFTs) from tau protein, impaired cholinergic signaling, accumulation of reactive oxygen species (ROS) and nitrogen species, upregulated glycogen synthase kinase 3 (GSK-3), downregulated cAMP response element-binding protein (CREB), and neuroinflammation (Sharma et al. 2019). Tau protein is normally responsible for binding of the microtubules, which in turn maintains the integrity of neuronal cells. When hyperphosphorylation of tau occurs, it loses its microtubules-binding ability (Kadavath et al. 2015). On the other hand, Ca^2+^/ calmodulin dependent kinase II (CAMKII) is one of the most common enzymes thar are responsible for hyperphosphorylation of tau protein (Sharma et al. 2019). CAMKII is activated by increased level of Ca^2+^ and calmodulin, and can be expressed on the cells in four isoforms; α, β, γ, and δ, where CAMKII-α and β are the most abundant isoforms in the brain, whereas CAMKII-γ and δ are expressed ubiquitously (Hoffman et al. 2013). Apart from its role in hyperphosphorylation of tau, increased expression of CAMKII can lead to downregulation of cAMP, which in turn downregulates protein kinase A (PKA), CREB, and brain-derived neurotrophic factor (BDNF) (Mika et al. 2015), which are critical for neuronal and cognition functions (Abdallah et al. 2020).

Lipopolysaccharide (LPS) is used for induction of neuroinflammation models in-vitro and in-vivo (Domínguez-Rivas et al. 2021). With excessive inflammatory conditions, microglial cells release pro-inflammatory cytokines such as tumor necrosis factor-alpha (TNF-α), interleukin-6 (IL-6), and IL-1β (Batista et al. 2019). This events can result in further hyperphosphorylation of tau and formation of NFTs and ultimately lead to neuritic injury and neuronal death (Sharma et al. 2019). In addition, LPS can increase the intracellular and mitochondrial Ca^2+^; the factor that cause upregulation of CAMKII and its downstream effectors (Silva et al. 2020). Loss of Ca^2+^ homeostasis has been implicated in the pathology of AD where Ca^2+^ dysregulation encourages the deposition of Aβ and NFTs; thereby, using calcium channel blockers may be beneficial neuroprotective agents (Tong et al. 2018).

Verapamil (VRP) is a cardioactive drug that was approved for treating several cardiac diseases owing to its voltage-dependent calcium channel (VDCC) blocking activity specifically the L type (Popović et al. 2020). It is worthy to mention that there is a circadian rhythm for the Ca^+2^ level, and the kinetic features of VRP are different meanwhile the daytime. Therefore, VRP chronotherapy should be taken into consideration (Popović et al. 2020).

From this point of view, we aimed to investigate the neuroprotective effect of VRP against LPS-induced neurotoxicity in experimental mice through anti-inflammatory effect and modulation of Ca^2+^-dependent genes involving CAMKII isoforms, PKA, CREB and BDNF. In addition, to elucidate whether the time of administration of VRP could affect the its efficacy.

## Materials and methods

### Drugs and chemicals

VRP was obtained from Abbott Pharmaceutical Company (Cairo, Egypt). LPS from *Escherichia coli* O26:B6, β-mercaptoethanol, and dimethylsulfoxide (DMSO) was purchased from Merck (USA). Phosphate buffered saline (PBS) was from Serana Europe GmbH (Germany). Tris-buffered saline with tween 20 (TBST) and bovine serum albumin (BSA) were from (BIO BASIC, Canada).

### Animals

Forty male albino mice aging 8–10 weeks and weighing 30–35 gm were obtained from The Egyptian Organization for Biological Products and Vaccines (Vacsera, Cairo, Egypt). The mice were adapted for about one month on tap water, veterinary diet, and day-night cycles. Animal housing and handling were according to the Guide for the Care and Use of Laboratory Animals (National Research Council) and the proposal was approved by Research Ethical Committee, Faculty of Science, Menoufia University, Egypt; approval number FGE421.

### Study design and disease induction

The mice were randomly divided into four different groups each with 10 mice; normal control, LPS only, morning VRP, and evening VRP. VRP groups were received the drug at a dose of 10 mg/ kg, i.p. daily for seven successive days prior to induction of neuroinflammation by LPS (Ponne et al. 2020). The normal control mice were received the vehicle; normal saline. The morning protection was at 9 am, whereas the evening protection was at 5 pm. Neuroinflammation was induced by LPS (750 μg/ kg, i.p.) dissolved in normal saline for another seven successive days (Zhao et al. 2019).

### Behavioral tests

#### Y-maze test

It is a Y letter designated maze of 3 identical arms; 30 × 5 × 12 cm divided by a fixed angle. It is used to evaluate the learning and discovering abilities that are controlled by the brain regions, which are largely affected by neuroinflammation. The 3 arms were labeled A, B, and C. A mouse was placed in one branch and allowed to discover the others over a period of 8 min. The correct manner is to enter different sequential arms; 3 different letters, in order to calculate the spontaneous alteration performance (SAP). The percent of spontaneous alteration (SA%) was calculated by the formula: $$\frac{number of alterations (NO of SAP)}{Total arm entries-2} x 100$$; where total arm entries = No. of entries to A + No. of entries to B + No. of entries to C (Wang et al. 2018a).

#### Pole climbing test

Pole climbing test is used to evaluate motor function that is controlled by the brain. The mice were placed at the upper part of a pole with up-warded head position, and the time required by the animal to turn its neck and reach the ground was recorded. For the mouse who fell directly, a 120 s were recorded (Ruan and Yao 2020).

### Sample collection

At the end of the experiment, the mice were sacrificed under light halothane anesthesia. Whole blood samples were collected in plain vacutainers to separate the sera for detection of inflammatory cytokines. Brain tissue were isolated, rinsed, split into pieces, and maintained in 80 °C for further analyses. The tissue for histopathological investigation were reserved in 7% formalin.

### Histopathological examination

Part of the tissue was stained by hematoxylin and eosin (H&E) to visualize neuroinflammation followed by quantification of the pyramidal cells. The other part was stained by Bielschowsky silver staining for visualization of phosphorylated tau protein (pTAU) (DeTure and Dickson 2019) followed by determination of the optical density of silver staining to generate quantitative measures. An appropriate microscope with a camera was used for imaging.

### Determination of microglia-activation markers

Specific monoclonal antibodies (mAbs) against CD11b and CD68 were purchased from Thermo Fisher Scientific (USA). CD11b was a surface staining, whereas CD68 was intracellularly stained. In brief, 100 μL of the tissue suspension was mixed with 3 μL of CD11b mAb followed by incubation in dark for 30 min. 2 mL of BD FACS lysing solution (BD Biosciences, USA) was mixed with the sample followed by another incubation for 15 min. Then, the samples were centrifuged with removal of the supernatant. Afterward, 2 mL PBS was used to wash the samples and then they were centrifuged again. For CD68, the procedures were the same but without addition of the mAb at the start. After discarding the last supernatant, 400 μL of eBioscience™ Foxp3 (Thermo Fisher Scientific, USA) was added to each sample and the samples were incubated for 15 min in dark. The samples were washed and centrifuged, and the precipitated pellets were resuspended in 100 μL PBS. Then, 4 μL of CD68 mAb was added with subsequent incubation. 2 mL PBS was mixed with the samples followed by centrifugation. The final step for both surface and intracellular staining were the resuspension of the pellets in 500 μL PBS. The analyses were performed via FACSCanto II cytometer (BD Biosciences, USA) and stainless samples were used as internal controls.

Ionized calcium-binding adaptor molecule 1 (Iba1) was also detected in the brain tissue of the mice by commercial ELISA kit purchased from MyBioSource (USA) according to the manufacturer’s protocol.

### Determination of inflammatory cytokines

The concentration of TNF-α, IL-6, and IL-1β was determined in the serum by using commercial ELISA kits purchased from MyBioSource (USA) according to the manufacturer’s instructions.

### Determination of mitochondrial membrane potential

Mitochondrial membrane potential (MMP) was determined immediately in the brain tissue by using image-iT™ TMRM reagent (Thermo Fisher Scientific, USA). This assay depends on using cell-permeant dye to label the active mitochondria with intact membrane potential. TMRM signals were detected with fluorescence microscopy Olympus BX41 (Olympus Co., Japan). Fluorescence intensity of the images was analyzed by ImageJ software to calculate corrected total cell fluorescence (CTCF) for each image by the formula: [(integrated density − (area of selected cell × mean fluorescence of background readings)] (Smulders et al. 2022).

### Determination of intracellular Ca^2+^

The level of intracellular Ca^2+^ was determined immediately in the brain tissue by using Fura-2 AM commercial Ca^2+^ indicator dye using fluorescence microscope. Pluronic™ F-127; low UV absorbance detergent was used to facilitate the AM ester solubilization. Calcium calibration buffer kit #1, zero and 10 mM CaEGTA was used to determine the dissociation constant (K_d_) of the fluorescent Ca^2+^ indicator; Fura-2 AM, and to construct a calibration curve to calculate the exact concentration of free Ca^2+^ for each studied group using FS5 spectrofluorometer (Edinburgh Instruments Ltd., UK). All kits were purchased from Thermo Fisher Scientific (USA).

### Western-blot analysis for CAMKII isoforms

The protein level of CAMKII α, β, γ, and δ was assayed by Western blotting. The extracted proteins from the brain tissue were determined by Bradford Protein Assay Kit (BIO BASIC, Canada). Then, the proteins were separated by sodium dodecyl sulfate–polyacrylamide gel electrophoresis (SDS-PAGE) and were transferred to polyvinylidene fluoride (PVDF) membrane. The membrane was blocked by TBST buffer and 3% BSA. Primary antibody (Santa Cruz Biotechnology, Inc., USA, CAT No. sc-5306) was used to detect CAMKII α, β, γ, and δ collectively at 51 kDa and 87 kDa two protein bands. HRP-conjugated secondary antibody (Novus Biologicals, USA) was then added. Afterward, a chemiluminescent substrate was applied to the blot according to the manufacturer’s recommendation. The signals were captured using a CCD camera-based imager. Bio-Rad ChemiDoc™ MP Imager was used to analyze the band intensity of the target protein against a control sample after normalization by β-actin.

### Determination of phosphorylated tau protein (pTAU)

The concentration of pTAU in the brain tissue homogenate was determined by commercial ELISA kit purchased from MyBioSource (USA) according to the supplier’s instructions.

### Determination of PKA, CREB, and BDNF

The expression level of PKA, CREB, and BDNF was determined by qRT-PCR. The sequence of the primers was designated by Primer-Blast. The designated primer sequences are displayed in Table 1 and were purchased from Macrogen (Korea). Total RNAs were extracted from tissue homogenate using RNeasy Mini Kit (QIAGEN, Germany). cDNAs for the extracted RNAs were obtained by EasyScript^®^ First-Strand cDNA Synthesis SuperMix (TransGen Biotech Co., China). Then, the amplification step was performed using QuantiTect^®^ SYBR^®^ Green PCR (QIAGEN, Germany) using StepOnePlus™ Real-Time PCR system (Thermo Fisher Scientific, USA). The expression level of the genes was expressed as relative copy number (RCN) and glyceraldehyde- 3-phosphate dehydrogenase (GAPDH) was used as a reference gene.

**Table 1:** Sequence of the used primers

Gene	Forward	Reverse
PKA	ACCCTATCACTCCCTGGCTC	GCACTAGCATTACGGTGGCT
CREB	AACGAAAGCAGTGACGGAGG	ACTCTGCTGGTTGTCTGCTC
BDNF	GCTGAAGGCGTGCGAGTATT	TGGTGGCCGATATGTACTCCT
GAPDH	TGCCAGGTGAAAATCGCGGA	CACTTCGCACCAGCATCCCT

*PKA* protein kinase, *CREB* cAMP response element-binding protein, *BDNF* brain-derived neurotrophic factor, *GAPDH* glyceraldehyde- 3-phosphate dehydrogenase

To ensure the activation, the protein level of phosphorylated CREB (P-CREB) against total CREB was determined by Western blotting as previously described for CAMKII isoforms. The Primary antibodies were from Santa Cruz Biotechnology, Inc., USA, CAT No. sc-81486 and sc-377154 to detect P-CREB and CREB; respectively, at 43 kDa. Additionally, the concentration of BDNF in the brain tissue was also determined by commercial ELISA kit (MyBioSource, USA) according to the supplier’s instructions.

### Determination of VRP level in the brain tissue

The concentration of VRP in the brain tissue was measured by HPLC. The brain tissue samples were homogenized with a fourfold excess volume of distilled water. To 1.5 mL of the homogenate, 1 mL methanol was added. The samples were shaken for 5 min and centrifuged at 8000 rpm for 15 min. The organic layer was separated and filtered using a 0.45 μm membrane filter (Millipore, Ireland) prior to HPLC acquisition.

The HPLC system was consisted of a liquid chromatograph [Agilent™ 1260 Infinity Quaternary HPLC System equipped with Agilent High Performance ALS (G1367E), Agilent Quaternary Pump (G1311B/C), thermostat Agilent autosampler with reliable injections from 0.1 to 100 μL (G1329B), and Agilent fluorescence detector (G1321C) operated at 231 nm (excitation) and 318 nm (emission)]. The column used for HPLC was ZORBAX^®^ Eclipse XDB C_18_ column (100 mm × 3 mm with 3.5 μm particle size) from Agilent. The mobile phase was acetonitrile-25 mM phosphate buffer (pH 4.0), methanol and acetonitrile (25:25:50, vol/vol). The flow rate of the mobile phase was 1.0 mL/min. The limit for quantification was 80 ng/mL in brain tissue.

### Statistical analysis

The raw data were analyzed by One-way analysis of variance (ANOVA) followed by Tukey significant difference as a post hoc test using IBM^®^ SPSS^®^ Statistics version 22 software (IBM Corp., Armonk, NY, USA). *P* < 0.05 was considered statistically significant different. GraphPad Prism and Adobe illustrator were used for the figures.

## Results

### Behavioral tests

**Fig. 1:**
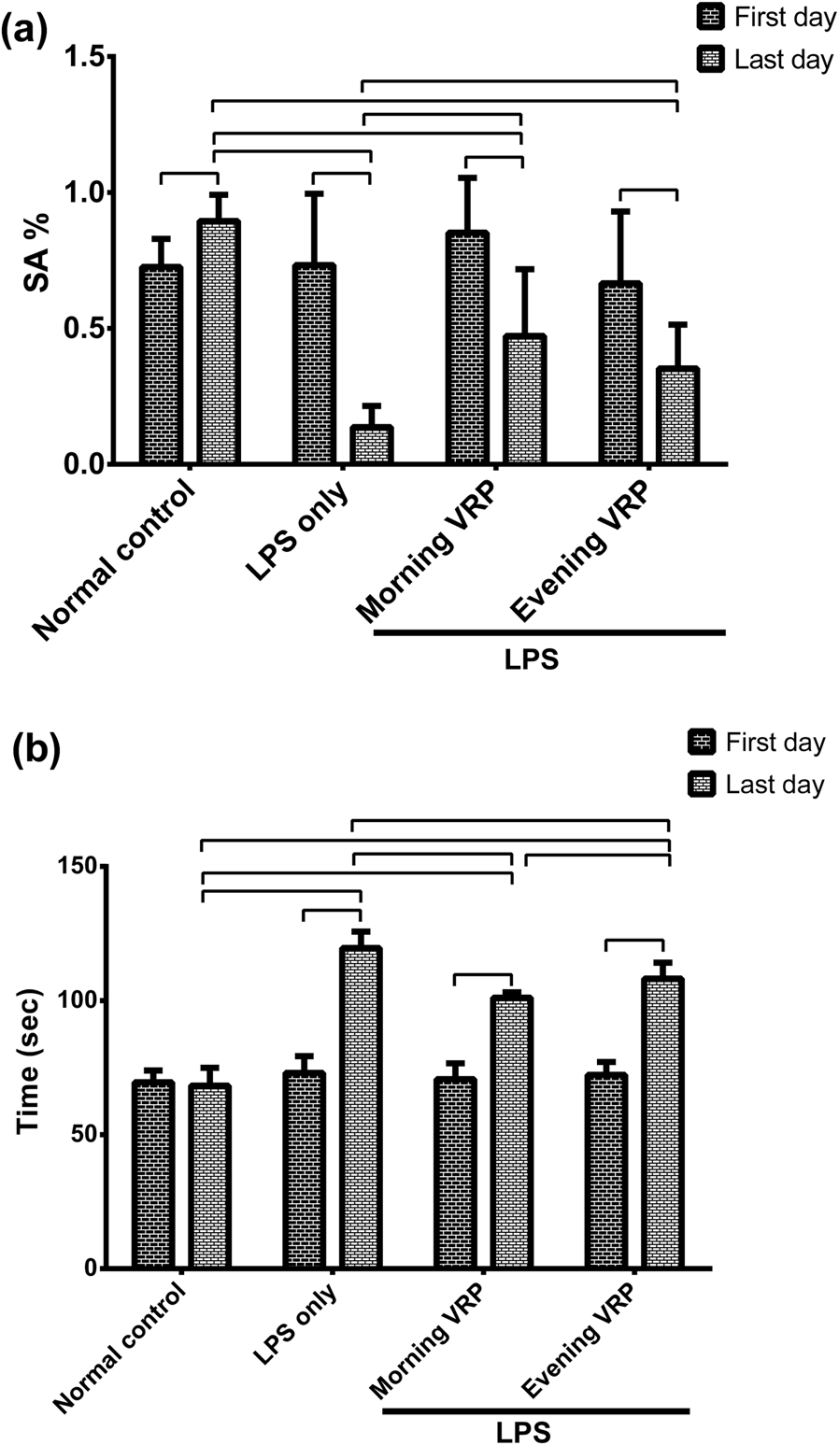
Effect of VRP on behavioral tests of the experimental mice. **a** Y-maze test and **b** Pole climbing test. Data are expressed as the mean ± SD and were analyzed using one-way ANOVA followed by Tukey post hoc test. Values were considered significantly different at *P* < 0.05. LPS: lipopolysaccharides, VRP: verapamil, SA%: percent of spontaneous alteration

Figure 1A represents SA% of the Y-maze test. There were no statistically significant differences between groups in the first day of the induction of the disease. In the last day of the induction, the LPS only group exhibited significant (*P* < 0.001) decline in SA% by 84.7% compared to normal control. The morning and evening pretreated groups with VRP showed significant (*P* < 0.001 and *P* = 0.022, respectively) increase in SA% by 2.4 and 1.5 folds; respectively, compared to the disease control. The SA% was in the following descendent order: normal control > morning VRP > evening VRP > LPS only. Regarding the within group comparison between the first and last day of the induction, all the studied groups showed significant decline in SA% in the last day compared to the start day with exception for the normal control group; it was significantly increased.

The results of pole climbing test were expressed in Fig. 1b. In the first day of the induction, there were no statistically significant differences between the groups. In contrast, the LPS control group showed significant (*P* < 0.001) increase in the time required to reach the ground by 75.1% compared to normal control in the last day. The morning and the evening VRP groups exhibited significant (*P* < 0.001) decline in the time by 15.4 and 9.5%; respectively, in comparison with the LPS control group. The time to reach the ground was in the following ascendent order: normal control > morning VRP > evening VRP > LPS only. Concerning within the group comparison, all the groups showed significant increases in the time in the last day compared to the first day.

### Histopathological examination

**Fig. 2:**
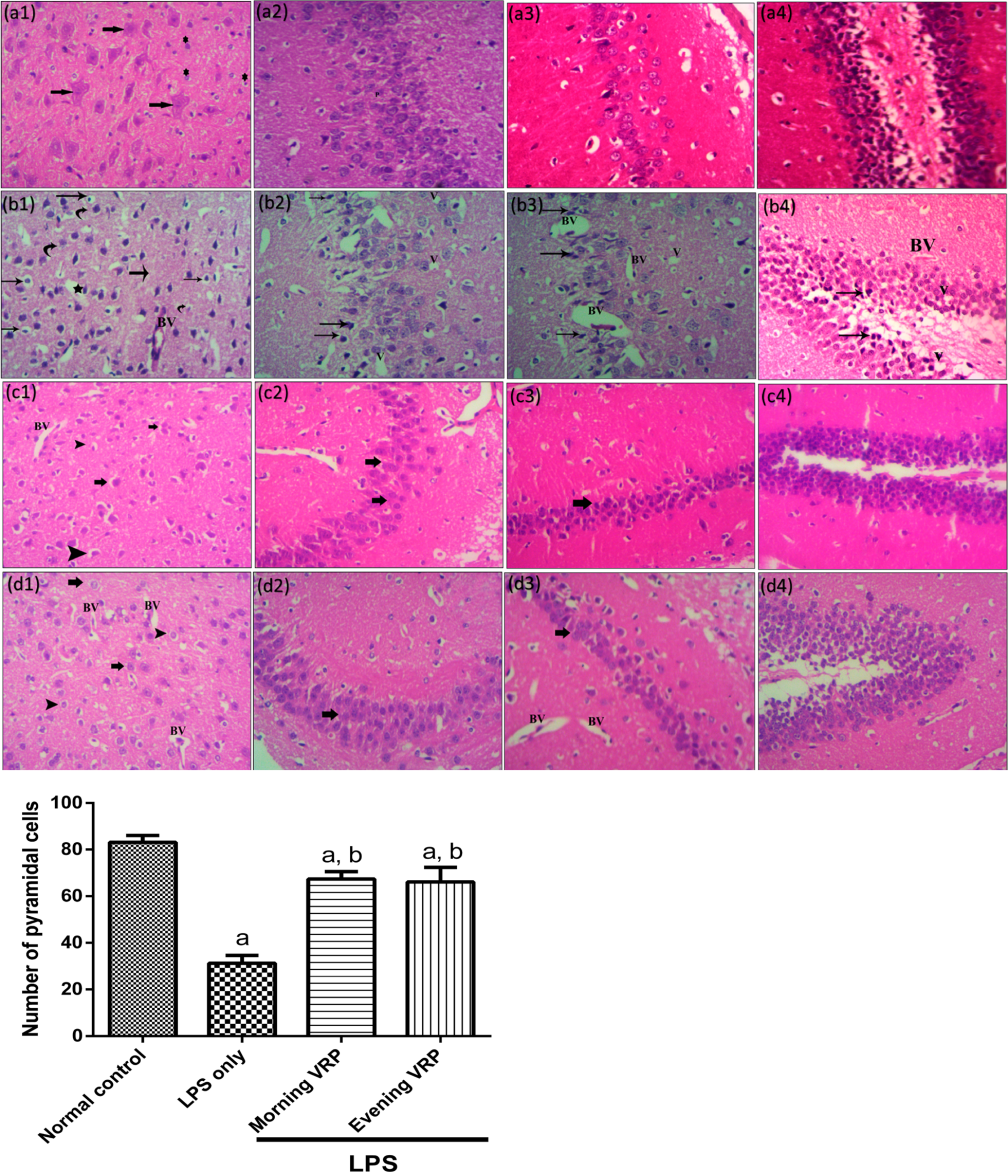
Effect of VRP on histopathological examination by H&E staining and quantitative measurement of pyramidal cells. **a** Normal control, **b** LPS only, **c** Morning VRP, and **d** Evening VRP. Four different sectors were examined; **1**: whole brain tissue, **2**: CA1 area, **3**: CA3 area, and **4**: dentate gyrus area. Data are expressed as the mean ± SD and were analyzed using one-way ANOVA followed by Tukey post hoc test. Values were considered significantly different at *P* < 0.05. a: significant *versus* normal control and b: significant *versus* LPS only. LPS: lipopolysaccharides, VRP: verapamil

The H&E staining sections were presented in Fig. 2. Normal control group (Fig. 2a1) showed normal brain architecture with normal pyramidal cells (arrow) and normal glial cells (star). The CA1 area of the brain (Fig. 2a2) shows normal hippocampal architecture with prominent pyramidal layer. CA3 hippocampal architecture (Fig. 2a3) and dentate gyrus (Fig. 2a4) were also normal. The photomicrograph of the LPS only group showed some irregular darkly stained pyramidal cells with pyknotic nuclei and surrounded by haloes (arrows). Other pyramidal cells were appeared with faintly stained cytoplasm and nuclei (curved arrow). There were also dilated congested blood vessels (BV) and extensive neuropil vacuolization (star) as showed in Fig. 2b1. The CA1 area (Fig. 2b2) showed numerous degenerated neurons with darkly stained nuclei (arrows) and many vacuolation (V). CA3 area (Fig. 2b3) and dentate gyrus (Fig. 2b4) displayed numerous degenerated neurons with darkly stained nuclei (arrows), many vacuolation, and multiple congested blood vessels. The morning VRP group (Fig. 2c1) and the evening VRP group (Fig. 2d1) showed normal pyramidal cells and granular cells (arrow) and some pyramidal cells are surrounded by haloes (arrow head). The evening group showed more congested blood vessels than the morning group. The morning and evening groups exhibited normal histological picture of CA1 (Fig. 2c2 and d2, respectively) and CA3 (Fig. 2c3 and d3, respectively) with normal vesicular nuclei of pyramidal cells (arrow) but the evening VRP still has some congested blood vessels in CA3 area. The dentate gyrus structure was nearly normal with its two blades in both groups (Fig. 2c4 and d4). For quantitative measures, the number of pyramidal cells was determined. The LPS significantly decreased the number of pyramidal cells (62.33%, *P* < 0.001) compared to normal control. Pretreatment with both morning and evening VRP significantly (*P* < 0.001) increased the pyramidal cells by 1.15 and 1.11 folds; respectively, compared to the LPS only group as presented in Fig. 2.

**Fig. 3:**
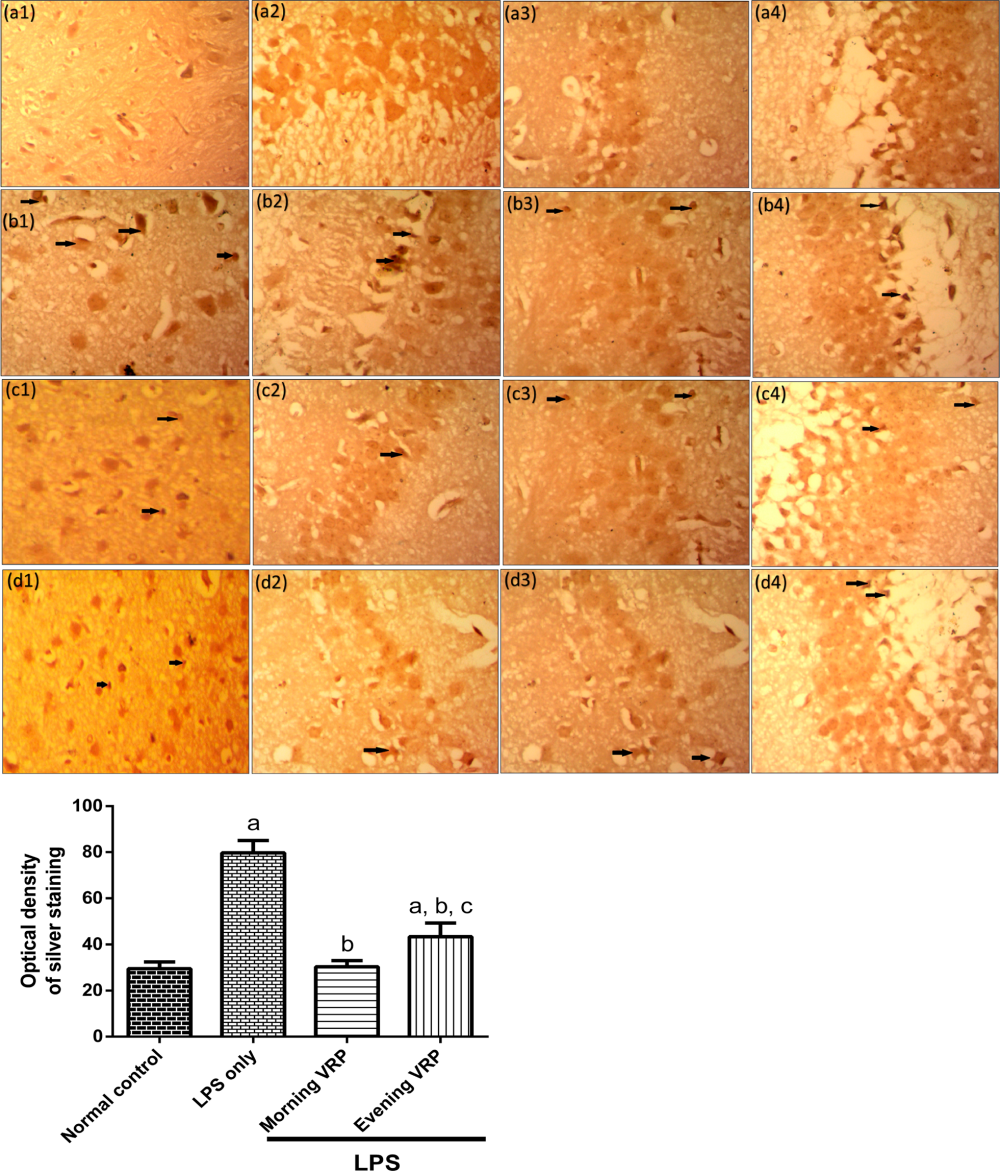
Effect of VRP on histopathological examination by Bielschowsky silver staining and quantitative measurement of optical density of the silver staining. **a** Normal control, **b** LPS only, **c** Morning VRP, and **d** Evening VRP. Four different sectors were examined; **1**: whole brain tissue, **2**: CA1 area, **3**: CA3 area, and **4**: dentate gyrus area. Data are expressed as the mean ± SD and were analyzed using one-way ANOVA followed by Tukey post hoc test. Values were considered significantly different at *P* < 0.05. a: significant *versus* normal control, b: significant *versus* LPS only, and c: significant *versus* morning VRP. LPS: lipopolysaccharides, VRP: verapamil

Regarding the Bielschowsky silver staining, the photomicrographs were presented in Fig. 3. Normal appearance of cerebral cortex (Fig. 3a1), CA1 (Fig. 3a2), CA3 (Fig. 3a3), and dentate gyrus (Fig. 3a4) was detected in the normal control group without any detectable microscopic abnormalities. The LPS only group showed multiple pTAU in different areas of the brain (Fig. 3b). The morning and evening VRP groups showed normal appearance of the brain tissue with very minimal amount of pTAU in different regions of the brain (Fig. 3c and d, respectively). For quantification, the optical density of the silver staining was determined. Comparing with the normal control, the LPS group showed significant (*P* < 0.001) increase in the optical density by 1.7 fold. In contrast, morning and evening VRP significantly (*P* < 0.001) lowered the optical density of silver staining by 61.85 and 45.54%; respectively, compared to the disease control as presented in (Fig. 3).

### Effect on microglia-activation markers

**Fig. 4:**
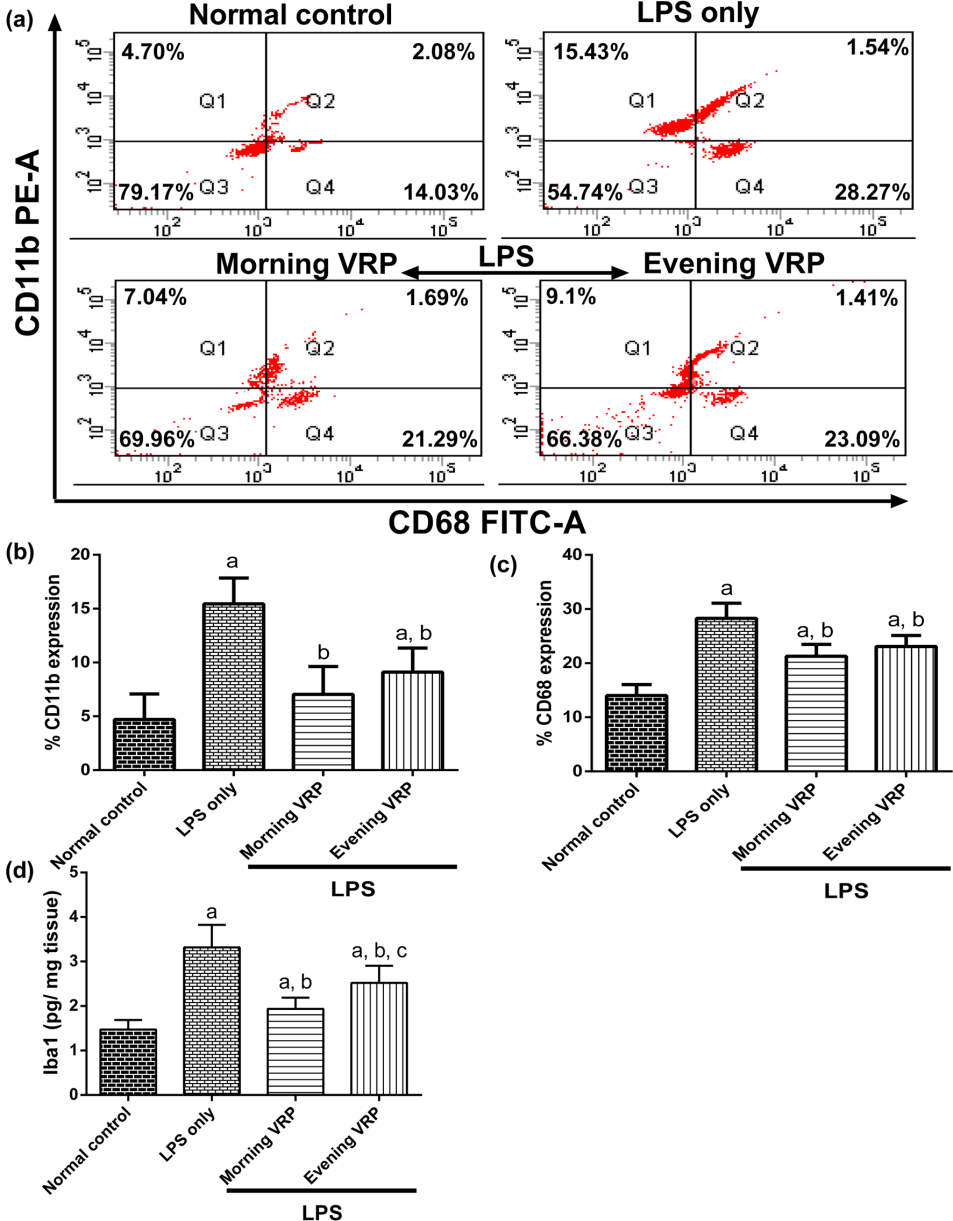
Effect of VRP on microglia-activation markers. **a** Dot plot flow cytometric analysis for one representative sample for each studied group, **b** Column chart for CD11b^+^ cells, **c** Column chart for CD68^+^ cells, and **d** Concentration of Iba1. For **b,** **c**, and **d** charts, data are expressed as the mean ± SD and were analyzed using one-way ANOVA followed by Tukey post hoc test. Values were considered significantly different at *P* < 0.05. a: significant *versus* normal control and b: significant *versus* LPS only, and c: significant *versus* morning VRP. LPS: lipopolysaccharides, VRP: verapamil, Iba1: Iba1: ionized calcium-binding adapter molecule 1

The LPS group showed significant (*P* < 0.001) increase in the number of the cells that are CD11b^+^ and CD68^+^ and also in the concentration of Iba1 (2.28, 1, and 1.25 folds, respectively) compared to normal control. The morning VRP pretreated group revealed significant (*P* < 0.001) decline in CD11b^+^, CD68^+^, and the level of Iba1 by 54.33, 24.68, and 41.33%; respectively, in comparison with the LPS only group. In the same manner, the evening pretreated mice showed significant (*P* = 0.001, *P* = 0.004, *P* < 0.001, respectively) drop in the markers by 41.01, 18.3, and 24.06%; respectively compared to the disease control group as presented in Fig. 4.

### Effect on inflammatory cytokines

**Fig. 5:**
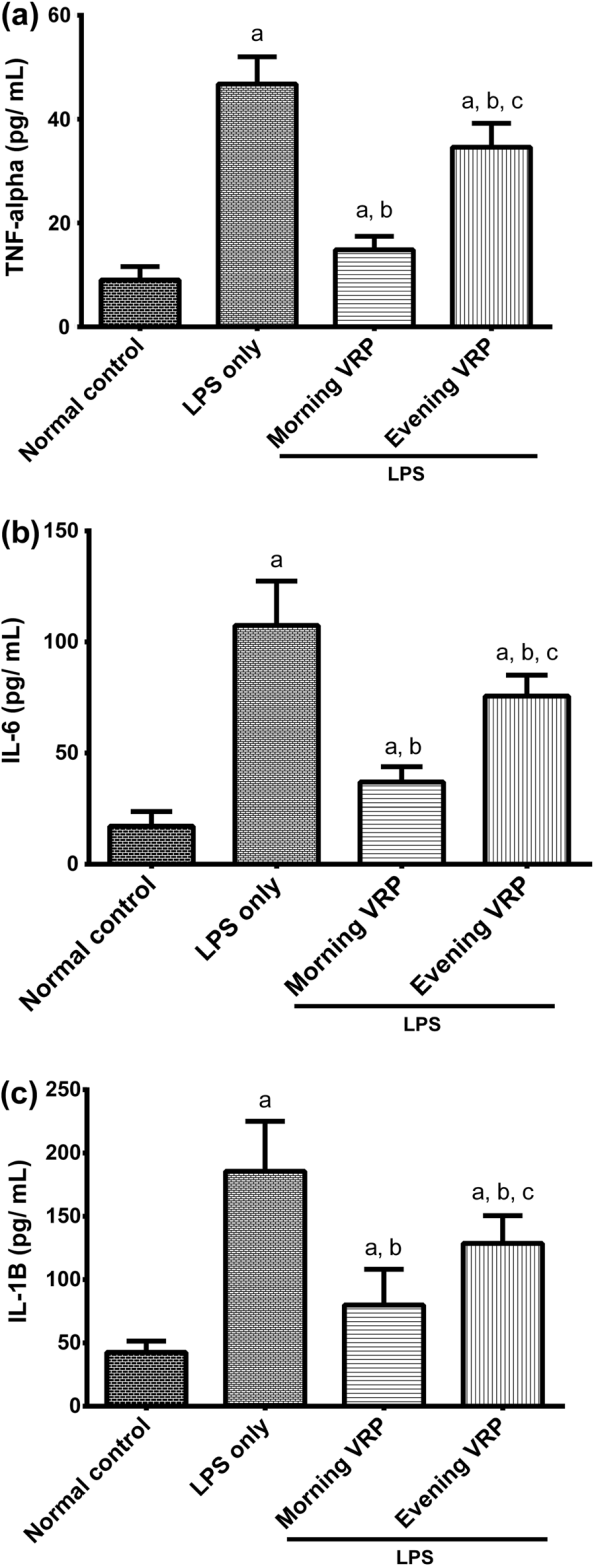
Effect of VRP on inflammatory cytokines. **a** TNF-α, **b** IL-6, and **c** IL-1β. Data are expressed as the mean ± SD and were analyzed using one-way ANOVA followed by Tukey post hoc test. Values were considered significantly different at *P* < 0.05. a: significant *versus* normal control, b: significant *versus* LPS only, and c: significant *versus* morning VRP. LPS: lipopolysaccharides, VRP: verapamil, TNF-α: tumor necrosis factor-alpha, IL-6: interleukin 6, IL-1β: interleukin 1 beta

Figure 5 shows that there was a significant (*P* < 0.001) increase in the concentration of TNF-α, IL-6, and IL-1β in the LPS group by 4.2, 5, and 3.1 folds; respectively, compared to normal control. In contrast, pretreatment of the mice with VRP whether in the morning or in the evening showed significant (*P* < 0.001) drop in the concentration of these cytokines by 69.18, 64.87, and 54.48%; respectively, for the morning VRP and by 26.11, 28.65, and 25.12%; respectively, for the evening VRP compared to the LPS only group.

### Effect on mitochondrial membrane potential

**Fig. 6:**
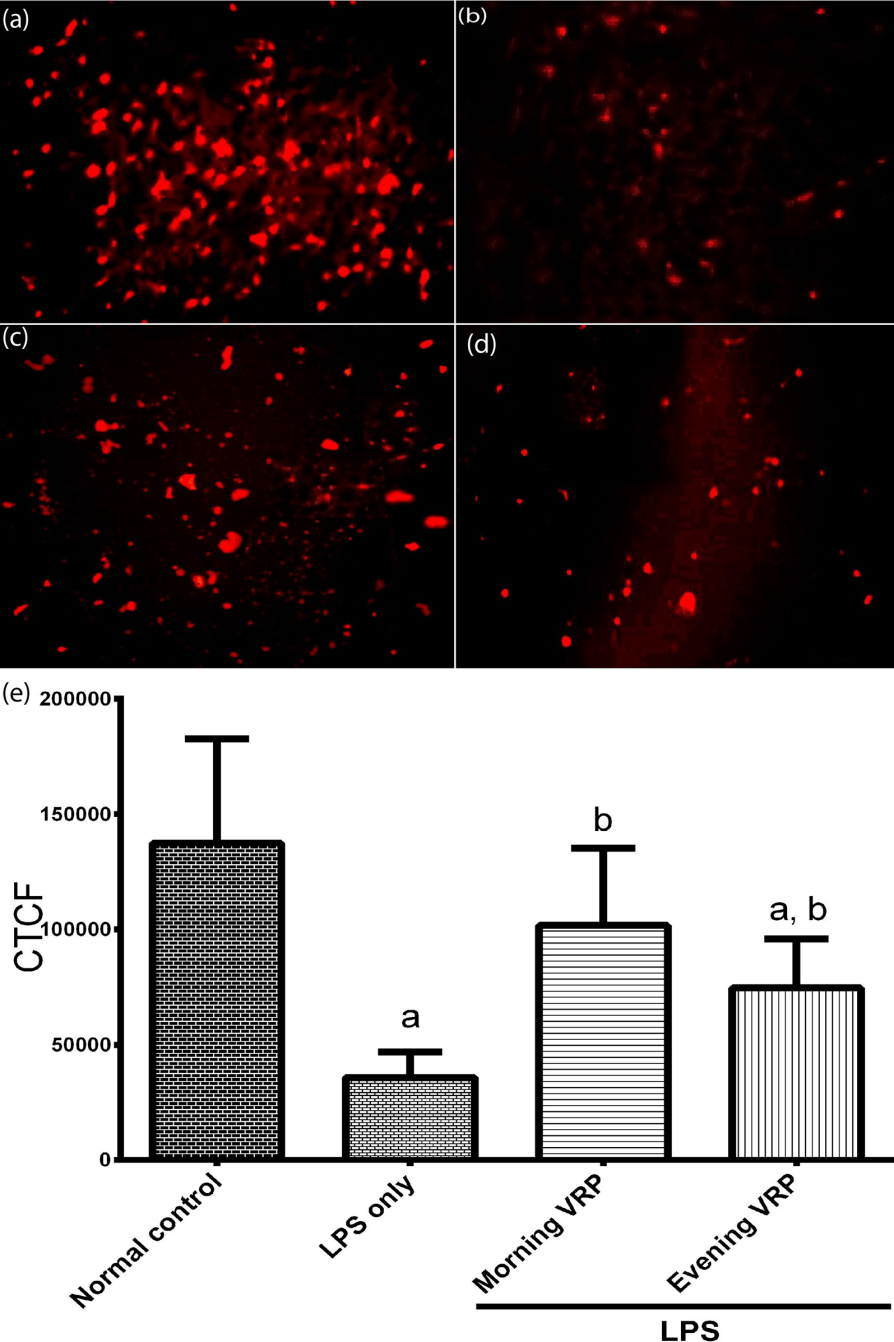
Effect of VRP on mitochondrial membrane potential. Representative fluorescent images from **a** Normal control, **b** LPS only, **c** Morning VRP, and **d** Evening VRP. **e** CTCF, data are expressed as the mean ± SD and were analyzed using one-way ANOVA followed by Tukey post hoc test. Values were considered significantly different at *P* < 0.05. a: significant *versus* normal control and b: significant *versus* LPS only. LPS: lipopolysaccharides, VRP: verapamil, CTCF: corrected total cell fluorescence

Figure 6a shows that the TMRM dye was localized inside the active mitochondria in the normal control group (bright red fluorescence). In contrast, the TMRM dye was diffused into the cytosol with less accumulation in the LPS control group (Fig. 6b). Both of morning and evening VRP groups have restored the accumulation of the TMRM cell-permeant dye and hence exhibited brighter fluorescence compared to the disease control group (Fig. 6c and d, respectively). These qualitative images have been quantified and expressed as CTCF (Fig. 6e). CTCF was significantly (*P* < 0.001) decreased in the LPS control group by 73.92% relative to the normal control, whereas the morning and evening VRP groups showed significant increase (1.8, *P* < 0.001 and one fold, *P* = 0.036, respectively) in the CTCF in comparison with the LPS only group.

### Effect on intracellular Ca^2+^

**Fig. 7:**
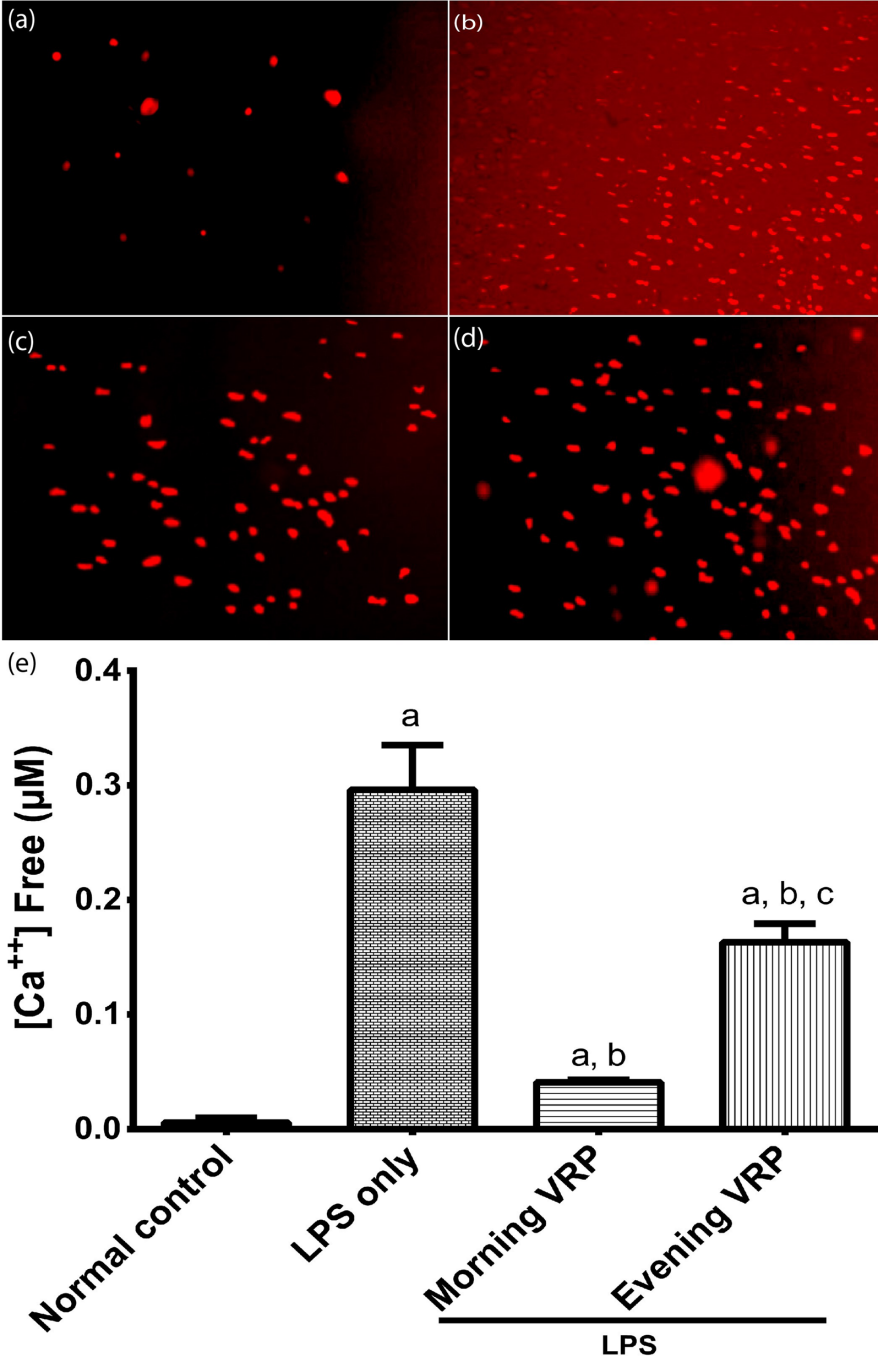
Effect of VRP on intracellular free Ca^2+^. Representative fluorescent images from **a** Normal control, **b** LPS only, **c** Morning VRP, **d** Evening VRP. **e** Calibrated intracellular free Ca^2+^, data are expressed as the mean ± SD and were analyzed using one-way ANOVA followed by Tukey post hoc test. Values were considered significantly different at *P* < 0.05. a: significant *versus* normal control, b: significant *versus* LPS only, and c: significant *versus* morning VRP. LPS: lipopolysaccharides, VRP: verapamil

Normal control group showed the lowest staining with Fura-2 AM dye and therefore reflected minimal fluorescence as in Fig. 7a. Induction with LPS increased the intracellular free Ca^2+^ and its staining with Fura-2 AM to very high level compared to normal control (Fig. 7b). Pretreatment of the mice with VRP in both groups have decreased the fluorescence intensity of the dye (Fig. 7c and d). After calibration and statistical analysis to generate a quantitative measure of free Ca^2+^, the LPS control group showed significant elevation (49 fold, *P* < 0.001) of the Ca^2+^ concentration compared to normal control. Morning and evening VRP groups significantly lowered (86.14 and 44.9%; respectively, *P* < 0.001) the intracellular free Ca^2+^ concentration compared to LPS control group (Fig. 7e).

### Effect on CAMKII isoforms

**Fig. 8:**
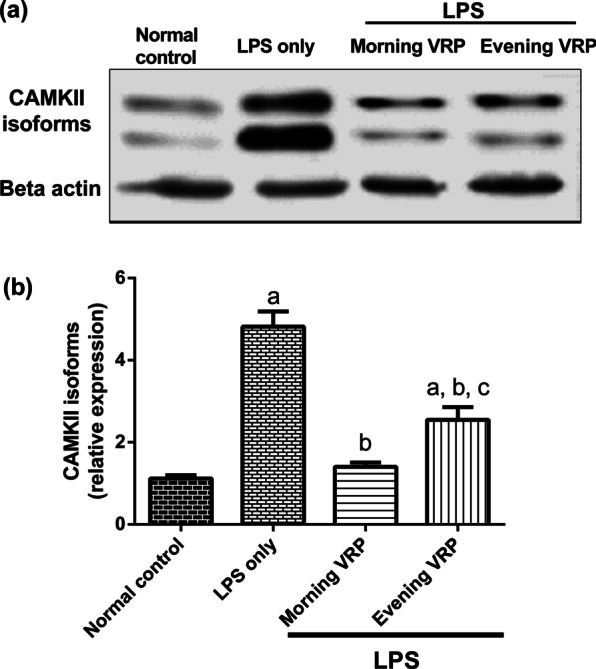
Effect of VRP on CAMKII isoforms. **a** Band intensity of CAMKII α, β, γ, and δ isoforms for one representative sample for each studied group normalized against β-actin. **b** Column chart for relative expression of CAMKII isoforms, data are expressed as the mean ± SD and were analyzed using one-way ANOVA followed by Tukey post hoc test. Values were considered significantly different at *P* < 0.05. a: significant *versus* normal control, b: significant *versus* LPS only, and c: significant *versus* morning VRP. LPS: lipopolysaccharides, VRP: verapamil, CAMKII: Ca^2+^/ calmodulin dependent kinase II

Figure 8 shows that the LPS group significantly (*P* < 0.001) upregulated CAMKII isoforms by 3.3 folds compared to normal group. The morning and evening VRP groups significantly (*P* < 0.001) downregulated CAMKII isoforms by 70.91 and 47.08%; respectively, in comparison with the LPS only group.

### Effect on pTAU

**Fig. 9:**
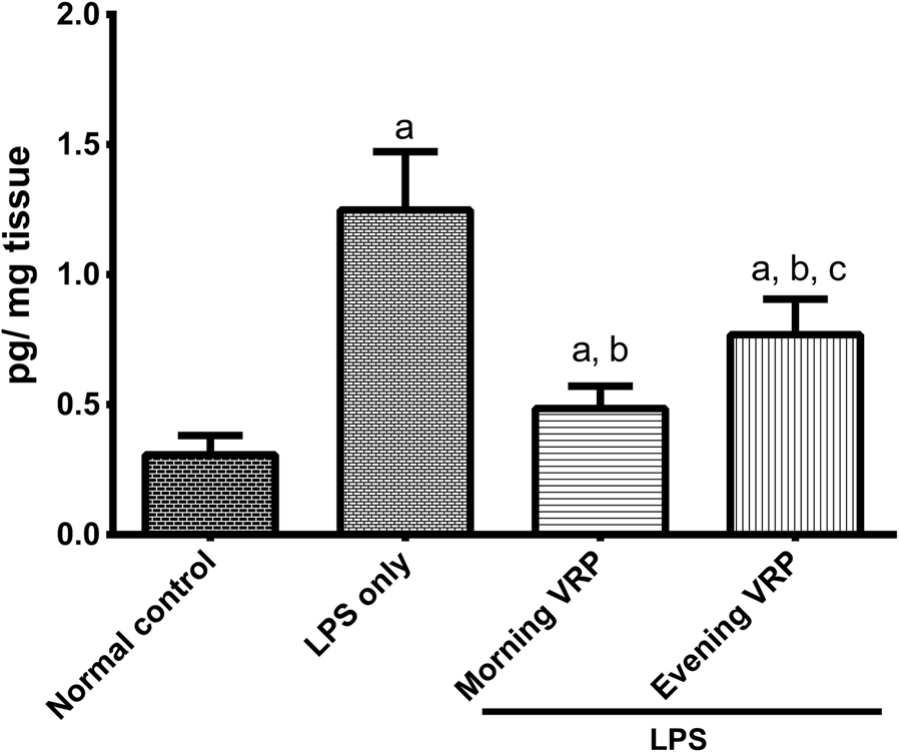
Effect of VRP on PTAU. Data are expressed as the mean ± SD and were analyzed using one-way ANOVA followed by Tukey post hoc test. Values were considered significantly different at *P* < 0.05. a: significant *versus* normal control, b: significant *versus* LPS only, and c: significant *versus* morning VRP. LPS: lipopolysaccharides, VRP: verapamil

The LPS control group significantly (*P* < 0.001) induced hyperphosphorylated tau protein by 3 folds compared to normal control. Relatively to the LPS control, protection of the mice with VRP in the morning and in the evening significantly (*P* < 0.001) lowered pTAU by 61.11 and 38.4%; respectively as presented in Fig. 9.

### Effect on PKA, CREB, and BDNF

**Fig. 10:**
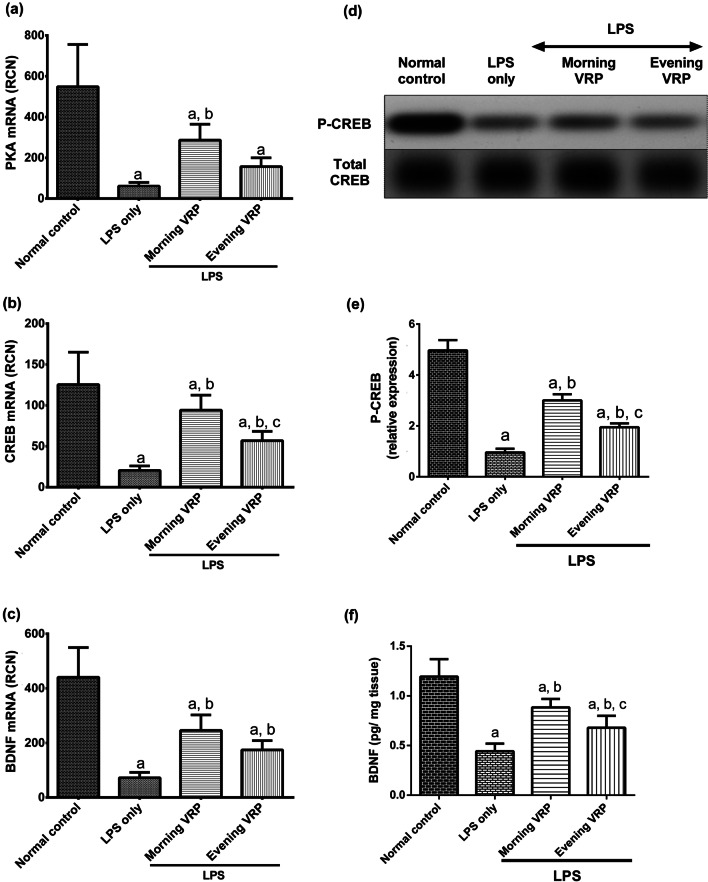
Effect of VRP on PKA, CREB, and BDNF. **a** Expression level of PKA, **b** Expression level of CREB, **c** Expression level of BDNF, **d** Band intensity of P-CREB for one representative sample for each studied group normalized against total CREB, **e** Column chart for relative expression of P-CREB, and **f** Concentration of BDNF. Data are expressed as the mean ± SD and were analyzed using one-way ANOVA followed by Tukey post hoc test. Values were considered significantly different at *P* < 0.05. a: significant *versus* normal control, b: significant *versus* LPS only, and c: significant *versus* morning VRP. LPS: lipopolysaccharides, VRP: verapamil, PKA: protein kinase A, CREB: cAMP response element-binding protein, P-CREB: phosphorylated CREB, BDNF: brain-derived neurotrophic factor

Figure 10 shows that the disease control group showed significant (*P* < 0.001) decline in the expression level of PKA, CREB, and BDNF by 88.87, 83.66, 83.68%; respectively, compared to normal control. Pretreatment of the mice with VRP in the morning and in the evening significantly elevated the expression level of these genes by 3.7, 2.4, and 3.6 folds (*P* < 0.001); respectively, for the morning VRP and by 1.5, 1.4, and 1.7 fold (*P* = 0.005 and *P* = 0.006 for CREB and BDNF); respectively, for the evening VRP relatively to LPS control group with exception for the PKA gene; the evening VRP showed non-significant increase (*P* = 0.251) as presented in Fig. 10.

To ensure the activation of the target genes, the protein level of P-CREB and BDNF was determined. Administration of LPS significantly decreased the concentration of P-CREB and BDNF (80.72 and 66.65%; respectively, *P* < 0.001) compared to normal group. In contrast, pretreatment with morning VRP showed significant increases in the level of these proteins (2.13 and 1.2 fold, *P* < 0.001) in comparison to LPS only. Similarly, the evening VRP also decreased the concentration of P-CREB and BDNF relatively to LPS group (1.03 fold and 70.13%, *P* < 0.001 and *P* < 0.003, respectively).

### Level of VRP in the brain tissue

**Fig. 11:**
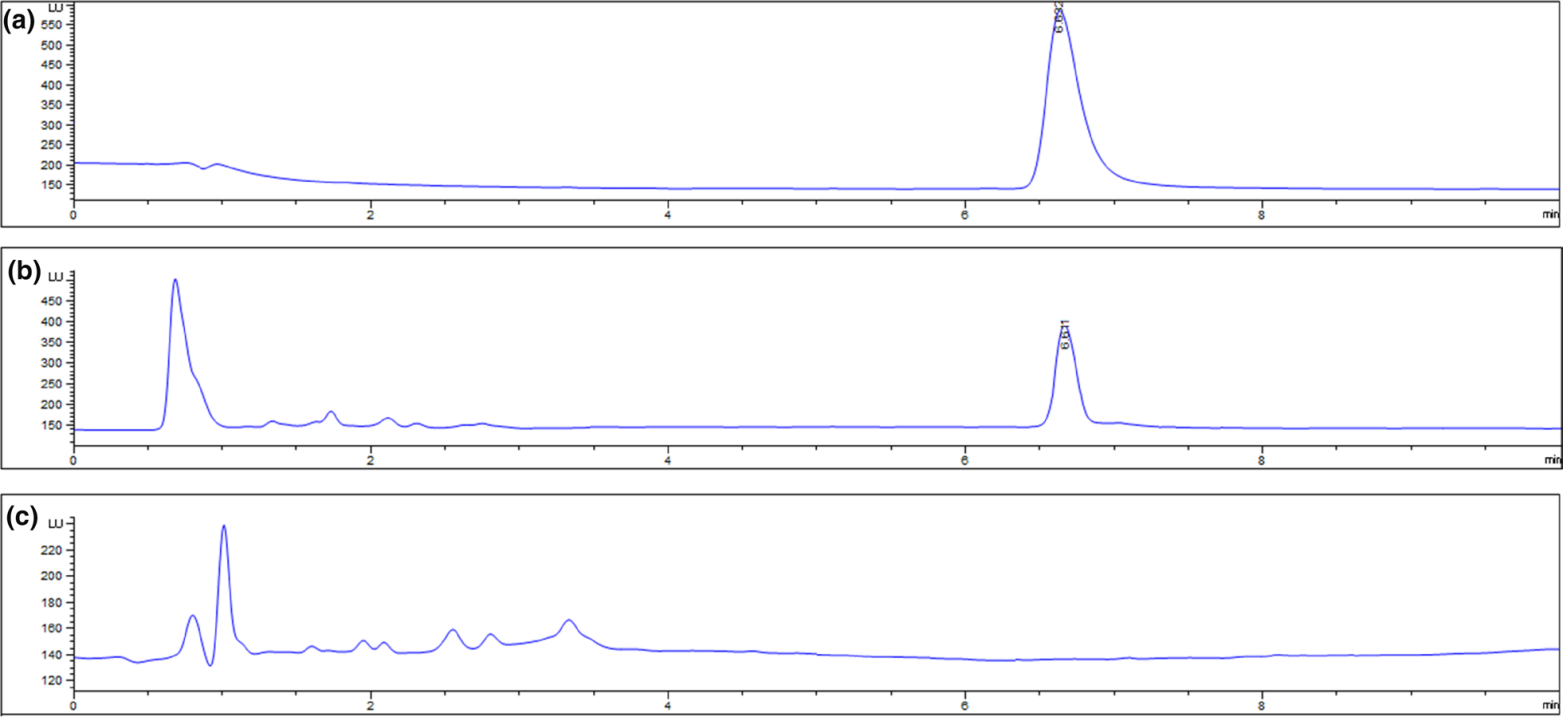
Chromatogram of detected level of VRP in the brain tissue of the experimental mice. Representative samples from **(a)** standard VRP, **(b)** morning and evening VRP, and **(c)** normal control; placebo

The measurement of VRP in the brain tissue homogenate revealed a concentration of 9.42 µg.mL^−1^ and 4.35 µg.mL^−1^ in morning and in the evening samples; respectively, as shown in Fig. 11.

## Discussion

In this study, the LPS control group showed elevated level of CD11b^+^ cells, CD68^+^ cells, Iba1, inflammatory cytokines, free Ca^2+^, pTAU, and CAMKII isoforms with low level of active mitochondria and downregulated PKA, CREB, and BDNF. These measured parameters were reflected by the distorted histopathological findings by H&E and silver staining and the negative impact was obvious in the behavior of the mice; their SA% was impaired and required long time to reach the ground in the pole climbing test.

Neuroinflammation is one of the main mechanisms of LPS. It happens due to stimulation of toll-like receptor 4 (TLR4), which is expressed by the neurons, resulting in the release of neurotoxic substances such as nitric oxide and free radicals (Calvo-Rodríguez et al. 2017). CD11b, CD68, and Iba1 are markers for the activated microglia; the cells that are highly implicated in neuroinflammation and they are activated in response to systemic injection of LPS. Earlier studies have documented a relation between AD and activation of microglia. The activated microglia release various inflammatory cytokines including TNF-α, IL-6, and IL-1β (Uff et al. 2022; Alster et al. 2020). This can explain the high population of CD11b^+^ and CD68^+^ cells, and consequently the elevated cytokines in the LPS group. Our results are in line with others that found LPS can induce microglia activation and elevation of CD11b, CD68, and Iba1 (Batista et al. 2019; Domínguez-Rivas et al. 2021; Steven et al. 2017; Dong et al. 2019) and with other studies, which reported the ability of LPS to elevate the cytokines and initiate neuroinflammation (Batista et al. 2019; Domínguez-Rivas et al. 2021; Dong et al. 2019; Meng et al. 2019).

Inasmuch as calcium homeostasis and MMP are intercalated with each other and play crucial role in the pathogenesis of AD, it was important to investigate these hallmarks. Preceding study has been demonstrated the oxidative stress-inducing property of LPS, which in turn dysregulates the MMP (Tripathi et al. 2017). Another study reported that the increased free Ca^2+^ can result in mitochondrial dysfunction and impaired membrane potential (Silva et al. 2020). This can elucidate the decreased MMP by LPS injection. Regarding the increased intracellular free Ca^2+^, it was suggested to be mediated through transient receptor potential cation channel (TRPV2), which promotes mobilization of Ca^2+^ from the intracellular and the extracellular stores. Moreover, it could be attributed to the activated TLR4 that triggers the release of neuro-excitatory substances that affect Ca^2+^ pool in the neurons (Calvo-Rodríguez et al. 2017). Our findings are in agreement with others that demonstrated the increased free cytosolic Ca^+2^ in response to LPS (Silva et al. 2020; Calvo-Rodríguez et al. 2017; Meng et al. 2019).

CAMKII is highly distributed in the hippocampus and brain cortex and it is largely implicated in the neuroinflammation (Song et al. 2019a). CAMKII is a downstream effector of Ca^2+^; thereby, increased Ca^2+^ can  activate CAMKII (Meng et al. 2019). In addition, the activation of CAMKII is provoked by the inflammatory conditions (Qian et al. 2021). Activation of CAMKII can activate other signaling pathways that are ultimately cause neuronal death (Song et al. 2019a). LPS was found to activate CAMKII isoforms, which could be attributed to the increased Ca^2+^ and the released inflammatory cytokines. Other studies have also reported the activation of Ca^2+^/ CAMKII by LPS (Meng et al. 2019; Song et al. 2019a; Qian et al. 2021). On the other hand, kinases enzymes such as CAMKII are involved in hyperphosphorylation of tau protein (Sharma et al. 2019). Nevertheless, activated microglia and inflammatory microenvironment can increase pTAU (Batista et al. 2019). Other studies suggested that LPS-induces taupathy through activation of mitogen-activated protein kinase (MAPK)/ c-Jun N-terminal kinases (JNK) signaling pathway (Song et al. 2019a; Yang et al. 2014). Our results are consistent with others that indicated the ability of LPS to induce taupathy (Wang et al. 2018a, 2018; Song et al. 2019a; Yang et al. 2014) and this was confirmed by the silver staining in our study.

BDNF plays important role in neuronal integrity and neurogenesis and it is subjected to control by various signaling pathways including CREB signaling (Abdallah et al. 2020). It is well known that the activated cAMP activate PKA, which is the upstream activator for CREB, which in turn upregulates BDNF (Xue et al. 2016). The relationship between cAMP/PKA and CAMKII signaling pathways is not fully understood and still needs much more investigations. However, Mika et al. reported that upregulation of CAMKII can result in downregulation of cAMP and the downstram target genes (Mika et al. 2015). These evidences can explain the downregulated PKA, CREB, and BDNF by LPS administration. These outcomes also come in line with other findings reported the supression of these genes by LPS (Yu et al. 2018; Wei et al. 2015). Undoubtedly, neurotoxicity induced by LPS will negatively affect the behavioral tests. Our results are consistence with other studies indicated the effect of LPS on different behavioral tests (Wang et al. 2018a; Tripathi et al. 2017; Ano et al. 2019).

While Ca^2+^ is playing an important role in learning and memory functions; action potential; and synaptic transmission, Ca^2+^ dysregulation is largely contributed to unfavorable events such as cell death, autophagy, neurodegenerative diseases including AD (Calvo-Rodriguez et al. 2020). Imbalanced Ca^2+^ can lead to mitochondrial damage, impaired synaptic signaling, production of ROS, neuronal cell death, and ultimately cognitive loss. Consequently, calcium channer blockers may represent promising neuroprotective agents (Popović et al. 2020). Based on these evidences, our results indicated that both of the morning and evening VRP-pretreated mice showed low CD11b^+^ cells, CD68^+^ cells, Iba1 level, inflammatory cytokines, free Ca^2+^, pTAU, and CAMKII isoforms with high activated mitochondria and upregulated PKA, CREB, and BDNF in comparison with the untreated diseased mice. These results were in consistence with the performed histopathological findings. The percent of spontaneous alteration was enhanced and the time in the pole climbing test was decreased, which reflects a neuroprotective effect of VRP.

Suppression of the activated microglia by VRP could be ascribed to its ability to inhibit the release of superoxide, nitric oxide, and inflammatory cytokines (Liu et al. 2011). Several studies reported the anti-inflammatory effect of VRP on different cell lines and in-vivo models by blocking the inflammatory mediators (Han et al. 2021; Ahmed et al. 2021; Song et al. 2019b) resulting in suppressed microglia. This suggests the anti-inflammatory action as one of the underlying neuroprotective mechanisms of VRP (Liu et al. 2011). Concerning the MMP and as we stated before, the impaired mitochondria could be a consequence of increased Ca^2+^, thus, after blocking of VDCC by VRP and decreased the free Ca^2+^, the mitochondria were restored. Our findings are in line with others that demonstrated VRP can enhance the mitochondrial functions due to inhibition of mitochondrial phospholipase, which contributes to the swelling of mitochondria and modulation of Ca^2+^ (Ponne et al. 2020; Jangholi et al. 2020). Other studies also attributed the neuroprotective action of VRP to the blockage of Ca^2+^ entry to the neurons (Liu et al. 2011; Maniskas et al. 2016; Darwish and Dessouky 2015), which supports our outcomes.

To the best of our knowledge, this study is the first to elucidate the effect of VRP on the overall isoforms of CAMKII. Undoubtedly, blocking of VDCC has led to decreased free Ca^2+^ and in turn decreased CAMKII. VRP can also inhibit transforming growth factor beta 1 (TGF-β1), which was found to trigger CAMKII (Zeng et al. 2021). Our results are in agreement with previous studies reported that VRP can downregulate CAMKII (Zeng et al. 2021; Gao et al. 2021; Mooney et al. 2015). Fortunately, suppression of CAMKII together with the restored mitochondria and anti-inflammatory effect decreased the level of pTAU. Town et al. suggested that blocking of VDCC by VRP decreases the activity of calpain protease that responsible for cleavage of p35 to p25; in turn, downregulates cyclin-dependent kinase 5 (Cdk5), which also involved in phosphorylation of tau protein (Town et al. 2002). Our conclusions are in harmony with other earlier works reported damping of pTAU by VRP (Town et al. 2002; Melone et al. 2018).

**Fig. 12:**
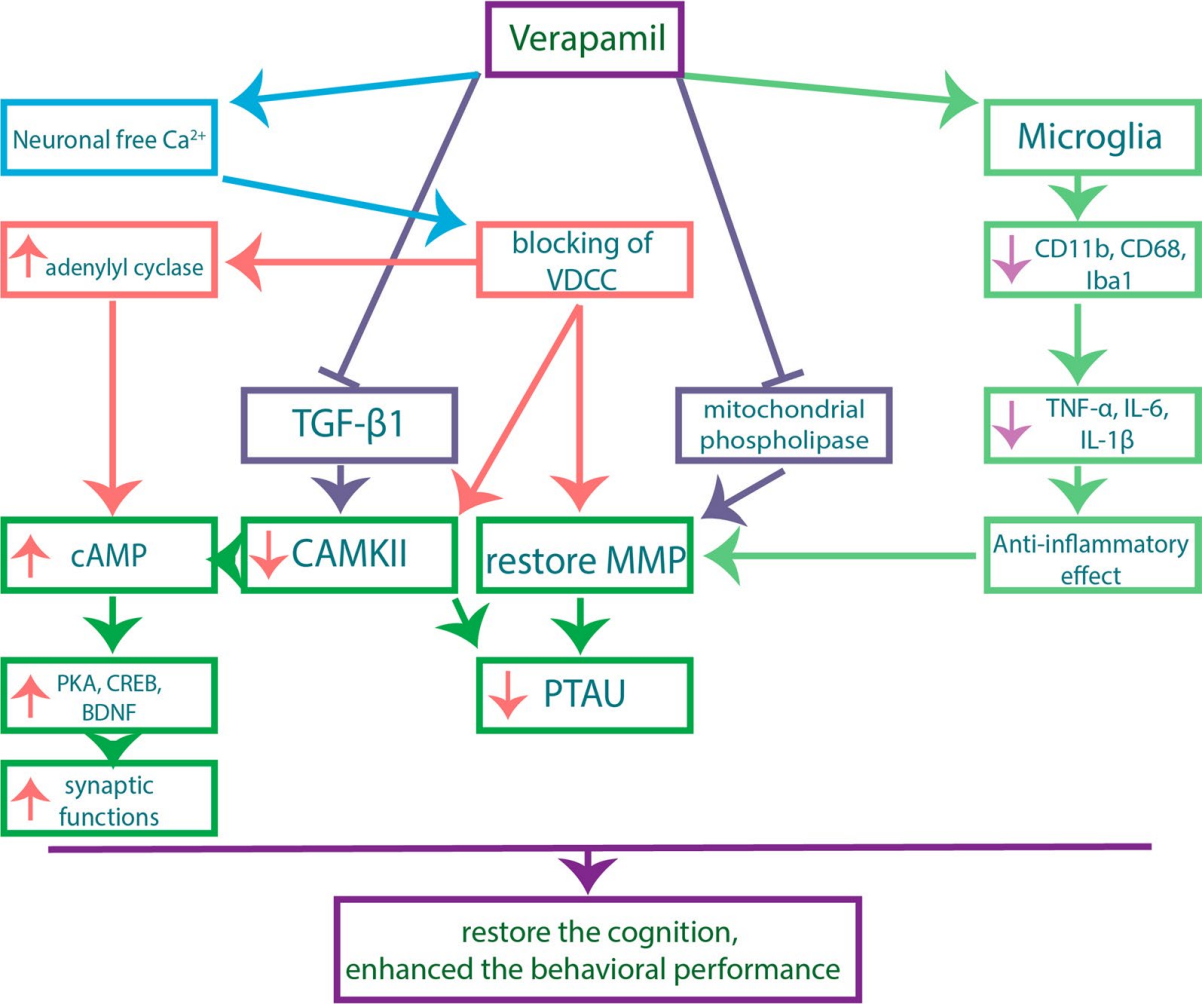
Possible neuroprotective mechanisms of VRP. Iba1: ionized calcium-binding adaptor molecule 1, TNF-α: tumor necrosis factor-alpha, IL-6: interleukin-6, IL-1β: interleukin 1β, TGF-β1: transforming growth factor beta 1, VDCC: voltage-dependent calcium channel, MMP: mitochondrial membrane potential, PTAU: phosphorylated tau protein, CAMKII: Ca^2+^/ calmodulin dependent kinase II, PKA: protein kinase A, cAMP: cyclic adenosine monophosphate, CREB: cAMP response element-binding protein, BDNF: brain-derived neurotrophic factor

Kusama et al. revealed that there is a reverse relationship between the intracellular free Ca^2+^ and the level of cAMP (Kusama et al. 2015). Moreover, Bergantin et al. indicated that blocking of VDCC by VRP increases the activity of adenylyl cyclase, which responsible for the production of cAMP (Bergantin et al. 2013). Consequently, these clues can explain the upregulated PKA, CREB, and BDNF in our study. Ponne et al. preceeded us in reporting that VRP can increase the expression of CREB and BDNF due to inhibition of Ca^2+^ (Ponne et al. 2020). Upregulation of these genes is substantial for neuroprotection because they greatly affect synaptic functions and; thereby, their elevation can restore the cognition. Therefore, VRP enhanced the behavioral performance of the pretreated mice and our findings are in line with several studies revealed the improved behavioral tests by VRP (Ponne et al. 2020; Ahmed et al. 2021; Giménez De Béjar et al. 2017). Figure 12 summerizes the possible neuroprotective mechanisms of VRP.

On one hand, some of the biological functions are subjected to a daytime variation and that called circadian rhythm, the factor that could affect the disease state and even concentration of the taken medications. Consequently, circadian abnormalities are largely contribute to neurological disorders (Lim et al. 2017). On the other hand, chronotherapeutics is defined as the field that focuses on proper timing of drug administration based on the circadian rhythm to obtain best efficacy and minimal side effects (Coulson et al. 2018). Less is known about the proper timing of drug administration for patients with AD, but based on the circadian rhythm of the Ca^2+^, we evaluated whether the morning or evening VRP is better. Our findings revealed that the morning VRP-pretreated mice exhibited better response in the behavioral tests, superior results of the measured parameters, and higher level of the drug in the brain tissue of these mice as indicated by the HPLC analysis. This could be explained by some conclusions offered by Lim et al., who reported that AD patients exhibited diurnal changes, compared to normal individuals, in terms of some genes’ expression and epigenetic modifications such as DNA methylation and histone acetylation (Lim et al. 2017). Another study by Singh et al. indicated that AD patients exhibit less morning motor activity compared to their evening activity (Singh et al. 2010). In addition, a previous work reported that the Ca^2+^ level is slightly higher in later time of the day compared to the morning level (Ridefelt et al. 2012). Similarly, our results are in concordant with Hla et al., who reported the chronokinetics of VRP where the morning plasma level of VRP and its active metabolite; norverapamil, was higher than the evening level and he attributed this findings to the factors that could affect the metabolism of VRP (Hla et al. 1992). These outcomes may elucidate why morning VRP showed better neuroprotective profile compared to the evening protection.

## Conclusion

VRP revealed a multilevel of neuroprotection against LPS-induced neurotoxicity through regulation of Ca^2+^ homeostasis and related genes including CAMKII isoforms, PKA, CREB, and BDNF. Downregulation of CAMKII isoforms decreased pTAU, one of the hallmarks of AD. The anti-inflammatory effect of VRP also played a critical role in restoring the cognitive functions and enhancing the behavioral performance of the brain, which reflected in the behavioral tests. Moreover, the chronotherapy of VRP should be considered because of the circadian rhythm that affect the disease state and our results revealed that morning administration was much better than the evening therapy.

## Data Availability

Data are available upon reasonable request.

## Abbreviations

AD: Alzheimer’s disease
Aβ: Amyloid beta
NFTs: Neurofibrillary tangles
ROS: Reactive oxygen species
GSK-3: Glycogen synthase kinase 3
CREB: CAMP response element-binding protein
CAMKII: Ca^2+^/ calmodulin dependent kinase II
PKA: Protein kinase A
BDNF: Brain-derived neurotrophic factor
LPS: Lipopolysaccharide
TNF-α: Tumor necrosis factor-alpha
IL-6: Interleukin-6
IL-1β: Interleukin-1 beta
VRP: Verapamil
VDCC: Voltage-dependent calcium channel
DMSO: Dimethylsulfoxide
PBS: Phosphate buffered saline
TBST: Tris-buffered saline with tween 20
BSA: Bovine serum albumin
SA%: Percent of spontaneous alteration
H&E: Hematoxylin and eosin
pTAU: Phosphorylated tau protein
mAbs: Monoclonal antibodies
MMP: Mitochondrial membrane potential
CTCF: Corrected total cell fluorescence
K_d_: Dissociation constant
SDS-PAGE: Sodium dodecyl sulfate–polyacrylamide gel electrophoresis
PVDF: Polyvinylidene fluoride
RCN: Relative copy number
GAPDH: Glyceraldehyde- 3-phosphate dehydrogenase
TLR4: Toll-like receptor 4
TRPV2: Transient receptor potential cation channel
MAPK: Mitogen-activated protein kinase
JNK: C-Jun N-terminal kinases
TGF-β1: Transforming growth factor beta 1
Cdk5: Cyclin-dependent kinase 5
